# A Retrospective Chart Review of Skin Cancer Pattern and Clinical Outcomes Among Saudi Patients Visiting a Tertiary Care Hospital in Western Saudi Arabia From 1987–2016

**DOI:** 10.7759/cureus.20666

**Published:** 2021-12-24

**Authors:** Sattam Almalki, Abdullah M Almalki, Faris Allaf, Abdullah Alrougi, Al-Hasan H Al-Marzouki, Fayssal Farahat

**Affiliations:** 1 College of Medicine, King Saud Bin Abdulaziz University for Health Sciences College of Medicine, Jeddah, SAU; 2 Infection Prevention and Control, King Abdulaziz Medical City, Jeddah, SAU

**Keywords:** squamous cell carcinoma, basal cell carcinoma, melanoma, incidence, skin cancer

## Abstract

Background

Skin cancer is one of the most common cancers worldwide. However, limited studies have been conducted on this disease in Saudi Arabia. This study aimed to describe the prevalence, treatment modalities, and outcomes of skin cancer in a tertiary care hospital in western Saudi Arabia.

Methods

A retrospective review of the medical records of all Saudi patients visiting King Abdulaziz Medical City hospital in Jeddah between 1987 and 2016 was performed.

Results

In total, 132 patients were diagnosed with skin cancer during the study period, however, only 119 cases were analyzed because of missing information. The male-to-female ratio was 1.09:1. The age at diagnosis ranges from 16-94 (mean age: 63.3 years). The most common type of skin cancer was basal cell carcinoma, followed by squamous cell carcinoma and malignant melanoma. The most common anatomical site was the head and neck region. The most common form of treatment modality was surgery. Of the studied patients, 49.6% were cured, 20.2% were in remission, 12.6% relapsed, and 17.6% died.

Conclusions

This study showed an equal male to female ratio with variant cumulative incidence over the years. Surgery was the most common treatment modality and had the highest curative outcome. Primary care physicians should be probed further to raise awareness and screen their patients to ensure early detection of possible skin cancer.

## Introduction

Exposure to ultraviolet (UV) light, primarily from sunlight, is an important factor in causing skin cancer. Other causes include immunosuppression, use of tanning booths, and direct contact with certain chemicals [1]. The three common types of skin cancer are basal cell carcinoma (BCC), which is the most common; squamous cell carcinoma (SCC), which is more common in immunocompromised individuals; and malignant melanoma (MM), which is the least common but has the worst prognosis because of its high metastatic ability [1]. Prognosis depends on the type and other factors [2]. 

Several studies have addressed the incidence and prevalence of skin cancer globally. A study in Australia conducted in 1978-1987 reported the case of 8,651 patients with nonmelanoma skin cancer, with patients with BCC accounting for more than two-thirds of the patients (n = 5,803), followed by those with SCC (n = 2,309) [3]. In Singapore, from 1968 to 1997, BCC was the most frequently reported cancer type (n = 2,650), followed by SCC (n = 1,407) and then MM (n = 281) [4]. In the United States, more than three million patients with nonmelanoma skin cancer and 96,480 patients with MM are diagnosed per year [5,6]. According to the National Cancer Institute, melanoma incidence increases by 1.5% per year [6]. Furthermore, one in five Americans will have skin cancer throughout their lives [7]. In India, a study investigated 92 patients with skin cancer, where the most frequent cancer type was SCC (n = 40), followed by BCC (n = 30) and MM (n = 13) [8].

In Saudi Arabia, a study conducted in the southwest region in 2004 reported that BCC accounted for 41% of all cancer types, followed by SCC at 29%, Kaposi sarcoma (KS) at 18%, and MM at 4% [9]. A 20-year retrospective study among Saudi patients in the eastern region showed similar results [10]. Moreover, in the western region of Saudi Arabia, a study conducted in 2000-2010 found BCC to be the most common cancer type, followed by SCC and then MM [11]. In the southern region, a study conducted in 1987-1991 on 137 patients with skin cancer showed that the most common cancer type was SCC (41.6%), followed by BCC (36.5%) and MM (11.7%) [12]. The authors in this study explained increased SCC cases to the secondary SCC (29.8%) developed from osteomyelitis skin sinuses, keloids, and traumatic scars [12].

Although several aspects of skin cancer have been addressed and discussed in the literature globally, recent studies in Saudi Arabia are lacking. Additionally, more information is needed regarding the pattern of treatment modalities and outcomes of skin cancer in Saudi Arabia. Therefore, the present study aimed to describe the pattern of skin cancer and assess its treatment modalities and outcomes in a tertiary care hospital in western Saudi Arabia from 1987-2016.

## Materials and methods

Methods 

A retrospective review was performed using the medical records of patients with skin cancer visiting King Abdulaziz Medical City (KAMC) hospital in Jeddah between 1987 and 2016. Data was collected using a case report form. The data collection sheet included the following items: sex, age at diagnosis, nationality, type of skin cancer, metastasis, treatment, precancerous lesion, date of biopsy, the result of the biopsy, anatomical position, and outcome. All Saudi patients with skin cancer who were diagnosed between 1987 and 2016 were identified. Patients with tumor metastasis to the skin, B cell lymphoma/leukemia with secondary skin involvement, and skin involvement by primary breast carcinoma and non-Saudi patients were excluded. Patients were categorized into four groups based on the following outcomes: (1) cured, defined as being discharged from the clinic; (2) in remission, defined as still being followed up after treatment; (3) relapsed, and (4) died.

Statistical analysis

Data were analyzed using IBM SPSS Statistics for Windows, Version 25.0. Armonk, NY: IBM Corp. Categorical variables such as sex and skin type were described as frequencies. Continuous variables such as age were described as mean [standard deviation (SD)]. A chi-square test was performed for categorical variables, and one-way analysis of variance (ANOVA) was used to compare more than two group means. Multivariate regression analysis was performed to determine associated risk factors for worse outcomes (death/relapse) using odds ratio and 95% confidence interval. The level of significance was determined at a p-value less than 0.05. 

## Results

Between 1987 and 2016, 132 patients were diagnosed with skin cancer, however 119 cases only were included in the study. Data were missed on 13 cases who were excluded from the analysis. Among the studied patients (n=119), 52.1% were male (n=62); the male-to-female ratio was 1.09:1. The mean (SD) age at diagnosis was 63.3 (16.3) years, with age ranging from 16 to 94 years. The most common type of cancer was BCC (39.5%, n = 47); SCC was the second (27.7%, n = 33) and MM was the third (17.6%, n = 21), followed by dermatofibrosarcoma (DFS) (8.4%, n = 10), adenoid cystic carcinoma (ACC) (2.5%, n = 3), BCC/SCC (2.5%, n = 3), and KS (1.7%, n = 2). Prelesions were reported in 19.3% of the cases. Most of the prelesions were among patients with SCC (60.9%) followed by MM (21.7%). Almost half of the cases (49.6%) were cured, 20.2% were in remission (still undergoing follow-up at time of data collection), 12.6% relapsed and 17.6% died. Patients with BCC had the highest cure percentage (78.7%), and those with KS and MM had the highest mortality percentage (50.0% and 47.6%, respectively) (Table 1). 

**Table 1:** Skin cancer outcome according to demographic characteristics and histological type BCC: Basal Cell Carcinoma, SCC: Squamous Cell Carcinoma, MM: Malignant Melanoma, DFS: dermatofibrosarcoma, ACC: adenoid cystic sarcoma, KS: Kaposi Sarcoma

Variables		Skin cancer outcome, n (%)				P value
		Cure	Death	Remission	Relapse	
Sex	Male	28 (45.2)	11 (17.7)	17 (27.4)	6 (9.7)	0.19
	Female	31 (54.4)	10 (17.5)	7 (12.3)	9 (15.8)	
Age; mean (SD)		64.43 (15.5)	63.55 (18.1)	64.04 (16.9)	57 (16.1)	0.49
History of Prelesion		10 (43.5)	4 (17.4)	8 (34.8)	1 (4.3)	0.23
Histological type	BCC	37 (78.7)	3 (6.4)	5 (10.6)	2 (4.3)	0.001
	SCC	14 (42.4)	7 (21.2)	7 (21.2)	5 (15.2)	
	MM	4 (19)	10 (47.6)	6 (28.6)	1 (4.8)	
	DFS	2 (20)	0 (0)	4 (40)	4 (40)	
	ACC	1 (33.3)	0 (0)	0 (0)	2 (66.7)	
	BCC/SCC	1 (33.3)	0 (0)	2 (66.7)	0 (0)	
	KS	0 (0)	1 (50)	0 (0)	1 (50)	

The most common anatomical site of the skin cancer lesion was the head and neck region (58.1%), followed by the lower extremities (19.7%). Of the head and neck lesions, 57.4% and 22.1% were BCC and SCC, respectively. Almost half of the lower extremities lesions were MM (47.8%) followed by SCC (21.7%). Metastasis was reported in 28.6% (n=34) of all patients. Cancer types with reported metastasis were MM (76.2%), DFS (40.0%), ACC (33.3%), SCC (30.3%) and BCC (6.4%). Most cases with metastasis died (44.1%), 29.4% were in remission, 17.6% were in relapse, and 8.8% were cured (Table 2). The most common treatment modality was surgery (92.4%), followed by radiotherapy (33.6%) and chemotherapy (16.0%). The following combined treatment modalities were used for patient management: triple combined therapy (10.9%), surgery and radiotherapy (32.8%), surgery and chemotherapy (11.8%), and radiotherapy and chemotherapy (10.9%). 

**Table 2:** Skin cancer outcome according to anatomic site and history of metastasis

Variables		Skin cancer outcome, n (%)				P value
		Cure	Death	Remission	Relapse	
Anatomic site	Head and Neck	41 (60.3)	7 (10.3)	12 (17.6)	8 (11.8)	0.06
	Upper Extremity	5 (55.6)	2 (22.2)	1 (11.1)	1 (11.1)	
	Lower Extremity	8 (34.8)	8 (34.8)	4 (17.4)	3 (13.0)	
	Trunk Front	0 (0)	1 (20)	2 (40)	2 (40)	
	Trunk Back	2 (33.3)	1 (16.7)	3 (50)	0 (0)	
	Genitalia	2 (33.3%)	1 (16.7)	2 (33.3)	1 (16.7)	
	History of metastasis		3 (8.8)	15 (44.1)	10 (29.4)		6 (17.6)	0.001

Table 3 presents a multivariate regression analysis of factors contributing to death or relapse among the studied patients. Only metastasis and treatment with radiotherapy were the significant factors associated with increased risk of death or relapse [Odds Ratio (OR)= 5.56, 95% confidence interval (CI) = 2.06, 15.04; OR= 3.72, 95%CI= 1.40, 9.91, respectively) (Table 3). 

**Table 3:** Multivariate logistic regression analysis of factors contributing to death or relapse The binary outcome is death/relapse versus cure/remission. BCC: Basal Cell Carcinoma, SCC: Squamous Cell Carcinoma, MM: Malignant Melanoma, DFS: dermatofibrosarcoma.

Variable	Odds Ratio	95% Confidence Interval	P value
Age; >60 years	1.67	0.58, 4.79	0.34
Gender; Female	1.91	0.71, 5.17	0.20
Prelesion; Yes	0.63	0.16, 2.50	0.51
Metastasis; Yes	5.56	2.06, 15.04	0.001
Surgery; Yes	0.96	0.13, 7.17	0.97
Radiotherapy; Yes	3.72	1.40, 9.91	0.009
Chemotherapy; Yes	1.32	0.33, 5.30	0.70
Cancer type;			
BCC	ref	ref	ref
SCC	2.68	0.70, 10.32	0.15
MM	4.0	0.80, 20.12	0.09
DFS	3.88	0.44, 34.35	0.22

Table 4 summarizes the national studies conducted in Saudi Arabia with a comparison of their findings with our study findings.

**Table 4:** Comparison of skin cancer characteristics among studies conducted in Saudi Arabia M:F: male-to-female ratio, SD: standard deviation, NMSC: nonmelanoma skin cancer, BCC: basal cell carcinoma, SCC: squamous cell carcinoma, MF: mycosis fungoides, MM: malignant melanoma, CTCL: cutaneous T-cell lymphoma, HN: head and neck, T: trunk

Common site	M:F ratio and mean age ±SD (years)	Most common types	Total study years	Sample size	Area	Author
HN	1.09:1, 63.3 ±16.3	BCC:SCC:MM	29	119	Jeddah	Our study
HN	1.6:1, 62.2	BCC:SCC:KS	13	193	Baha	Almaghrabi (9)
HN	1.85:1, ---	BCC:SCC:MF	19	123	Dhahran	Aldawsari (10)
HN	2.1:1, 46:6	BCC:SCC:MF	10	106	Jeddah	Mufti (11)
HN	1.6:1, 61	SCC:BCC:MM	5	137	Asir	Bahmdan (12)
HN	1.6:1, 59 ± 19	BCC:SCC	13	279 (NMSC)	Riyadh	Alsalman (14)
HN	2.25:1, 60	BCC:SCC:MM	10	104	Taif	Alaboud (15)
HN	3:1, 59 ± 20	BCC:SCC:MM	9	94	Qassim	Alzolibani (16)
HN	1.4:1, 67.2	BCC:SCC	20	82 (NMSC)	Dammam	Alakloby (17)
T:HN	1.17:1, 53 ± 25	CTCL:SCC:BCC	5	98	Asir	Hafez (18)
HN	2.2:1, 60 ± 15	BCC:SCC:MF	11	202	Medina	Albasri (19)

**Figure 1:**
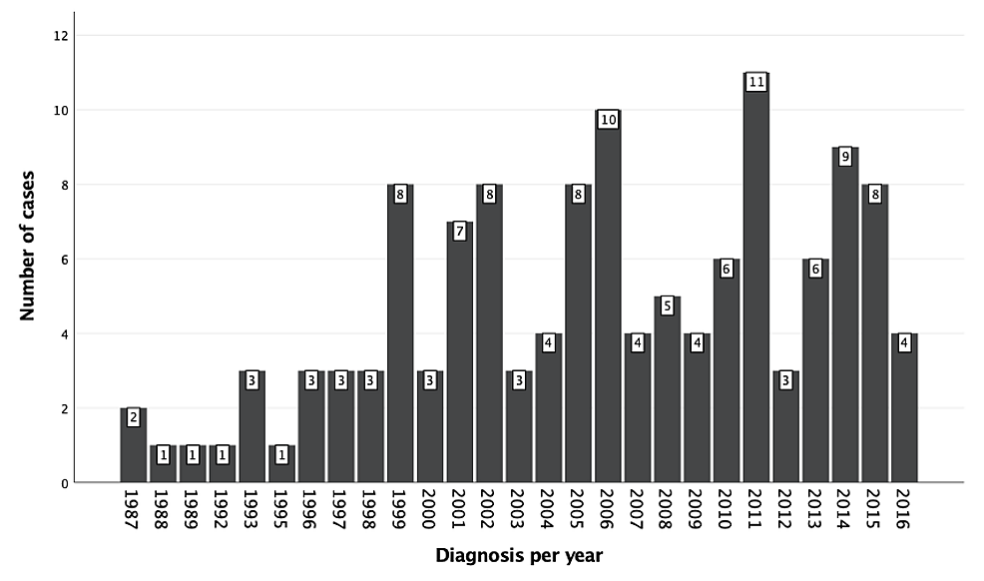
Yearly number of diagnosed skin cancer cases from 1987 - 2016.

Figure 1 depicts the yearly number of diagnosed skin cancer cases from 1987-2016. No consistent pattern has been shown. 

## Discussion

Our study findings are consistent with those of previous studies regarding the two most common types of skin cancer: BCC and the SCC [9-12,13-17]. The third common type varied among studies in different regions of Saudi Arabia, with KS being the third common cancer type in the southwestern region [18]. In this study, MM was the third most common cancer type (17.6%); however, its reported percentage also varies across studies (3.7%-10.3%) [9-12,14-15,17,18]. Mycosis fungoides (MF) has been excluded from the current study as it is a cutaneous T cell lymphoma, although it has been reported as the third common condition in other studies from the eastern and western regions of the country [10-11,19]. 

The overall occurrence of skin cancer in the current study was very low, consistent with other similar studies [9,11,14]. Our study revealed an almost equal incidence of skin cancer based on sex (1.09:1), whereas other studies have shown a higher male-to-female ratio, ranging from 1.17:1 to 3:1 [9-12,13-18]. This observed discrepancy may be attributed to the different levels of UV exposure. Men and women are covered with traditional clothes like "thoab and abaya", additionally skin tanning is not common among Saudi people [20]. However, further investigation into the degree of sun exposure by the two sexes in different regions is warranted. 

The overall mean age at diagnosis in our study is 63.3 (SD, 16.3) years, which is consistent with the findings of most studies conducted in Saudi Arabia [9,12,13-18], apart from one study in Jeddah, which reported a mean age of 46.6 years [11].

Our study reveals that the most frequently affected anatomical site was the head and neck, followed by the lower extremities. This is similar to that reported by other studies in Saudi Arabia, with the lower extremities being reported as the second most frequently affected site, especially in MM [9-12,13-17].

Prelesions were reported in 19.3% of patients in this study. Other studies have reported a prelesion in 29.8% and 38.2% of the studied patients [12,13]. However, our analysis showed no statistical significance regarding the treatment outcome and presence of a prelesion.

The majority of BCC (78.7%) were cured, compared to 42.4% of SCC, 33.3% of ACC, 20% of DFS, 19% of MM, and none of KS cases. The overall cure percentage among the studied patients was (49.6%). Remission was observed in one-fifth of the patients (20.2%) and mortality in 17.6%. However, recurrence (relapse) was reported in 12.6% of the studied patients. In the current study, the relatively high mortality can be attributed to the late diagnosis and intervention.

Metastasis was reported in 34 patients in our study, although it was not reported in other studies. About half of cases with metastasis were MM (47.1%) followed by SCC (29.4%). Importantly, only 8.8% of those with metastasis were cured compared to 65.9% cured among patients with no reported metastasis.

The most common treatment modality was the surgical excision of the lesion. This was consistent with the international patient management guidelines, which recommend surgery as the first-line treatment modality for BCC and SCC [19]. This study has shown that more than half of patients who undergone surgery cured (52.7%) compared to 27.5% among those with radiotherapy and 10.5%% among patients with chemotherapy treatment. 

The multivariate regression analysis identified metastasis and treatment with radiotherapy alone or in combination with other modalities as the significant predictors of worse outcomes represented by death or relapse. 

This study had several limitations related to missing data owing to its retrospective nature of the review of the patients’ medical records over a long period. The study may not represent the general population because of its single-center study design. However, KAMC is a tertiary care main referral hospital in the western region of Saudi Arabia. However, the small number of cases in this study is another limitation due to the overall low incidence of skin cancer in Saudi Arabia.

## Conclusions

In conclusion, the risk of skin cancer is almost equal between the male and female sexes and starts increasing after the age of 50 years. The most common type of skin cancer is BCC, followed by SCC and MM. The most common anatomical site was the head and neck region and then the lower extremities. The most common treatment choice for skin cancer lesions was surgery. The majority of BCC patients were cured, however about half of MM and a fifth of SCC cases died. 

Screening for skin cancer among high-risk populations should be increased; primary healthcare providers and family medicine physicians should be probed further to raise awareness among and facilitate screening for their patients. This will result in the early detection of skin cancer and improved treatment outcomes.
